# Combining Intravoxel Incoherent Motion Diffusion Weighted Imaging and Texture Analysis for a Nomogram to Predict Early Treatment Response to Concurrent Chemoradiotherapy in Cervical Cancer Patients

**DOI:** 10.1155/2021/9345353

**Published:** 2021-12-24

**Authors:** Xiaomin Zheng, Cuiping Li, Lufeng Zhang, Feng Cao, Xin Fang, Liting Qian, Jiangning Dong

**Affiliations:** ^1^Department of Radiation Oncology, The First Affiliated Hospital of University of Science and Technology of China, Hefei, China; ^2^Department of Radiation Oncology, Anhui No. 2 Provincial People's Hospital, Hefei, China; ^3^Department of Radiology, The First Affiliated Hospital of Anhui Medical University, Hefei, China; ^4^Department of Urology, Hefei BOE Hospital, Hefei, China; ^5^Department of Radiology, The First Affiliated Hospital of University of Science and Technology of China, Hefei, China

## Abstract

This study aimed to predict early treatment response to concurrent chemoradiotherapy (CCRT) by combining intravoxel incoherent motion diffusion weighted imaging (IVIM-DWI) with texture analysis (TA) for cervical cancer patients and to develop a nomogram for estimating the risk of residual tumor. Ninty-three cervical cancer patients underwent conventional MRI and IVIM-DWI before CCRT. We conducted TA using T2WI. The patients were allocated to partial response (PR) and complete response (CR) groups on the basis of posttreatment MRI. Multivariate logistic regression analysis on IVIM-DWI parameters and texture features was employed to filter the independent predictors and construct the predictive nomogram. Its discrimination and calibration performances were estimated. Multivariate analysis on the IVIM-DWI parameters showed that *D* and *f* were independent predictors (OR = 4.029 and 0.889, resp.; *p* < 0.05). However, the multivariate analysis on the texture features indicated that GLCM-correlation, GLRLM-LRE, and GLSZM-ZE were independent predictors (OR = 43.789, 9.774, and 23.738, resp.;*p* < 0.05). The combination of IVIM-DWI parameters and texture features exhibited the highest predictive performance (AUC = 0.975). The nomogram to identify the patients with high-risk residual tumors exhibited an acceptable predictive performance and stability with a C-index of 0.953. Decision curve analysis demonstrated the clinical use of the nomogram. The results demonstrate that *D*, *f*, GLCM-correlation, GLRLM-LRE, and GLSZM-ZE were independent predictors for cervical cancer. The nomogram combining IVIM-DWI parameters and texture features makes it possible to identify cervical cancer patients at a high risk of residual tumor after CCRT.

## 1. Introduction

As the fourth most commonly diagnosed type of cancer, cervical cancer globally ranks fourth among cancer-related deaths in female [1]. To treat locally advanced cervical cancer, concurrent chemoradiotherapy (CCRT) is suggested, and its recurrence was reported in 30% of cases with cervical cancer [2, 3]. Furthermore, given the tumor heterogeneity, it is unlikely that different types of cancer respond to a particular treatment in the same way [4]. Therefore, it is important to accurately predict early treatment response, which may significantly affect the prognosis. In addition, clinicians will be able to adjust treatment approaches in case of reliable biomarkers identifying patients in time, who are at a high risk of residual tumor.

Morphological changes can be observed by conventional magnetic resonance imaging (MRI). With the emergence of diffusion weighted imaging (DWI), characterization and detection of diseases improved, probing the diffusion of water molecules in biological tissue. Intravoxel incoherent motion DWI (IVIM-DWI), initially described by Le Bihan et al., was proposed to extend DWI using an increased number of *b* values [5, 6]. At low *b* values, data obtained are dominated by perfusion effects, while signal delay captured at high *b* values is mainly attributed to diffusion [7]. IVIM-DWI parameters can be used to monitor therapeutic alterations during CCRT, in addition to differentiating benign lesions from cervical cancer [8, 9]. In the previously mentioned studies, significant information is reported on both characterizations of carcinoma and treatment response by IVIM-DWI.

Texture analysis (TA), a series of mathematical methods, can assess the position of pixels and gray level intensity of an image. Texture features can be extracted by TA, assisting in measuring intralesional heterogeneity [10]. Previous reports showed that some pretreatment texture features, and the changes in texture features, were related to the outcome of various tumors [11–13]. T2-weighted imaging (T2WI) MRI has been widely used for the local staging evaluation of cervical cancer, providing important anatomical and functional information on the tumor as well as better tissue contrast. T2WI-based texture features have also been proven to provide potential predictive information for histological tumor differentiation, lymph node metastasis, and lymphovascular space invasion in cervical cancer [14, 15]. To date, no scholar has reported data predicting early treatment response for cervical cancer patients after CCRT using T2WI-based texture features.

The present research aimed to assess the function of IVIM-DWI parameters and texture features to predict early treatment response in patients with cervical cancer after CCRT. A nomogram was developed for the individualized prediction of cervical cancer patients at a high risk of residual tumor.

## 2. Materials and Methods

### 2.1. Patients

This retrospective study was conducted in accordance with the Declaration of Helsinki. The institutional Review Board of the First Affiliated Hospital of University of Science and Technology of China approved the study and waived the requirement for patients' informed consent owing to the retrospective and observational nature of this study (approval number: 2020-YXK-01). From January 2016 to January 2020, 93 cervical cancer patients admitted to our hospital were enrolled in the study. Inclusion criteria were as follows: (1) cervical cancer confirmed by pathology; (2) performing conventional MRI and IVIM-DWI less than two weeks before undergoing CCRT; (3) an acceptable image quality for diagnosis and measurement; (4) availability of clinical and pathological information. Exclusion criteria were as follows: (1) a history of undergoing radiation/chemotherapy; (2) other tumor diseases in the same period. Supplementary Figure 1 illustrated the process of patient selection.

### 2.2. Imaging Examination

All patients were given routine pelvic MRI and IVIM-DWI prior to CCRT, as well as pelvic MRI plain scan and contrast-enhanced MRI one month after CCRT. A 3.0 T MRI system (Signa HDxT, GE Healthcare, USA) was employed to conduct MRI. Patients were asked to lie in a supine position and have an empty urinary bladder. The parameters affiliated to MRI sequences were as follows: (1) axial fast spin-echo (FSE) T1WI: repetition time (TR)/echo time (TE), slice thickness, interslice gap, number of excitation (NEX), field of view (FOV), and matrix size were 500/7.5 ms, 4 mm, 2 mm, 2, 24 × 24 cm, and 256 × 256, respectively; (2) sagittal and oblique (perpendicular to the long axis of the cervical canal) FSE T2WI: TR/TE, slice thickness, interslice gap, NEX, FOV, and matrix size were 4200/68 ms, 4 mm, 2 mm, 2, 24 × 24 cm, and 320 × 256, respectively; (3) axial fat-suppressed T2WI: TR/TE, slice thickness, interslice gap, NEX, FOV, and matrix size were 4200/68 ms, 4 mm, 2 mm, 2, 24 × 24 cm, and 320 × 256, respectively. IVIM-DWI was acquired using the axial free-breathing single-shot spin-echo echo-planar imaging (SS-SE-EPI) sequence. The main parameters were as follows: *b* = 0, 20, 40, 60, 100, 200, 400, 800, 1200 s/mm^2^; TR/TE, slice thickness, interslice gap, NEX, FOV, and matrix size were 4000/77 ms, 4 mm, 1 mm, 6, 42 × 42 cm, and 160 × 96, respectively. The imaging duration was 7 min and 54 s.

### 2.3. Imaging Analysis

**Figure 1:**
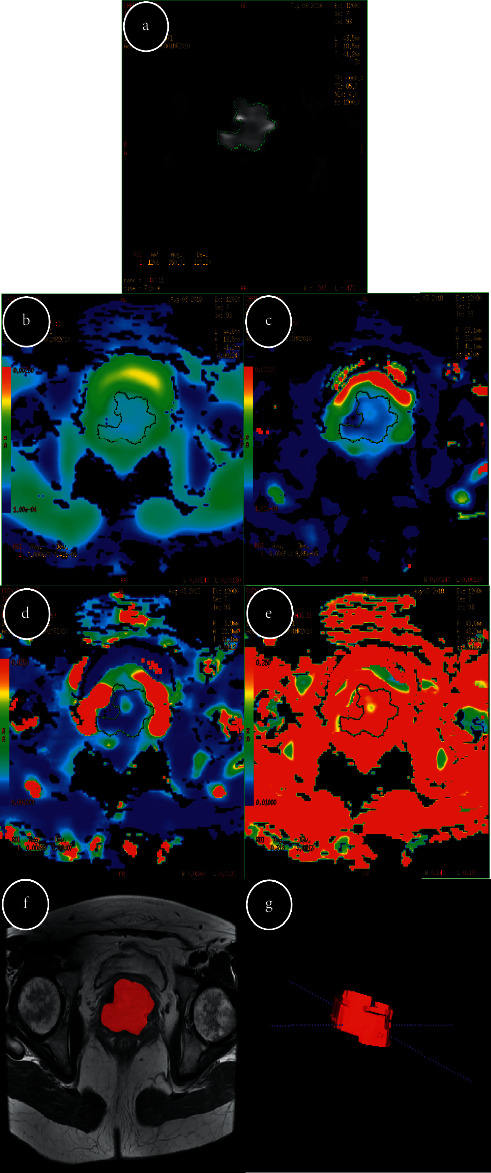
Example of manually drawing ROIs for cervical cancer. IVIM-DWI measurement (*b* = 1200 s/mm^2^) (a); each radiologist drew ROI-1 (5 mm^2^) three times to get the values on the maps of ADC, *D*, *D*^*∗*^, and *f*, respectively (b–e). Texture analysis, tumor segmentation in cervical cancer, and segmentation were performed on T2WI (f); by stacking up segmented ROIs slice-by-slice, VOI covering the entire tumor (g) was acquired. ROI: region of interest; IVIM-DWI: intravoxel incoherent motion diffusion weighted imaging; ADC: apparent diffusion coefficient; *D*: slow diffusion coefficient; *D*^*∗*^: fast diffusion coefficient; *f*: perfusion-related diffusion fraction; T2WI: T2-weighted imaging; VOI: volume of interest.

To carry out IVIM-DWI measurement, two radiologists (8 and 10 years of pelvic diagnostic experience, resp.), having no idea of clinicopathologic results, used FuncTool to create the region of interest (ROI) on a postprocessing workstation of GE AW 4.6. The ROI of IVIM-DWI was selected on the maximum transverse plane of each lesion (*b* value = 1200 s/mm^2^) to obtain the parameters of apparent diffusion coefficient (ADC), slow diffusion coefficient (*D*), fast diffusion coefficient (*D*^*∗*^), and perfusion-related diffusion fraction (*f*) (Figure 1). For each case, the ROI was drawn thrice to calculate the average value, avoiding necrosis, hemorrhage, or cervical canal as much as possible. Furthermore, each radiologist drew twice to get the values, and the intraclass correlation coefficient (ICC) was calculated to assess the interobserver agreement.

Regarding TA on T2WI, axial T2WI images were segmented using ITK-SNAP software. By stacking up segmented ROIs slice-by-slice, it was possible to acquire volumes of interest (VOIs) covering the entire tumor. Two radiologists reached an agreement on the chosen area of ROI. ROI was drawn to cover the whole lesion, including necrosis/hemorrhage (Figure 1). Then, 74 texture features, including 18 histogram features, 24 GLCM (gray level co-occurrence matrix) features, 16 GLRLM (gray level run length matrix) features, and 16 GLSZM (gray level size zone matrix) features, were extracted using a software package (Artificial Intelligence Kit Version 3.2.2, GE Healthcare) (Supplementary Table 1). Another radiologist with a five-year experience in gynecological imaging was included to analyze and delineate the tumor margins based on a subset of 25 randomly selected patients, thus assessing the interobserver agreement of texture features. Then, from ROI markings, the texture features were extracted and compared with those extracted from the markings of other radiologists.

### 2.4. CCRT and Early Treatment Response

All patients were treated by external beam radiotherapy (EBRT) combined with high dose-rate intracavitary brachytherapy (HDR ICBT). EBRT was given based on a daily dose of 1.8–2.0 Gy, 5 days/week, 25 or 28 times (total dose: 45–56 Gy). The volume of the EBRT was indicated by radiography before the therapy in accordance with the nodal status. The chemotherapy was given on day 1, namely, the commencement of radiotherapy, with concurrent administration of cisplatin/nedaplatin at 30−40 mg/m^2^, weekly. HDR ICBT was given with a dose of 30 Gy after the EBRT for three weeks. ICBT was given with a daily fraction of 5.5–6 Gy, twice a week, five fractions, with the dose of 27.5–30 Gy at point A (corresponding to the paracervical triangle along the medial edge of the broad ligament, where uterine vessels cross the ureter). The therapeutic regimens were adjusted in accordance with the health status of the patients.

After one month of CCRT completion, the response of early treatment was evaluated via MRI based on RECIST [16]: (1) complete response (CR) could be achieved when no residual tumor could be seen on MR images; (2) partial response (PR) could be achieved when the residual tumor's largest diameter was 30% smaller than the original size; (3) progress of disease (PD) was assessed when the longest diameter of the tumor increased by at least 20% compared with the pretreatment size; (4) disease as stable (SD) was considered when there was neither an adequate increase to qualify PD, nor an adequate decrease to measure PR. According to the criteria mentioned above, 58 patients were classified as CR and 35 as PR; no PD or SD was identified.

### 2.5. Statistical Analysis

The continuous data were presented with mean ± standard deviation (SD). The chi-square test and one-way analysis of variance (ANOVA) were employed to compare patients' clinical characteristics for categorical and continuous variables. The Mann-Whitney *U* test and ANOVA were used for feature selection, and the features with significant differences between the CR and PR groups (*p* < 0.05) were chosen for subsequent multivariate logistic regression analysis. The multivariate logistic regression analysis was adopted to estimate odds ratios (ORs) and 95% confidence intervals (CIs) of independent predictors and risk of outcomes. Receiver operating characteristic (ROC) curve analysis was employed to determine the predictive performance of IVIM-DWI, TA, and combined (IVIM-DWI with TA) models.

Independent predictors were applied to establish a nomogram for predicting the risk of residual tumor in cervical cancer patients after CCRT. To quantify the performance of prediction of the nomogram, a fivefold cross-validation was performed, a calibration curve was drawn, and the Brier score was calculated. Harrell's concordance index (C-index) was also measured. In brief, a C-index value >0.75 represents relatively satisfactory predictability. In addition, the decision curves were plotted for IVIM-DWI, TA, and combined models. ICCs were selected to assess the interobserver agreement with respect to the measurement of IVIM-DWI parameters and texture features, respectively (ICC > 0.8 indicates almost perfect agreement). We attempted to conduct statistical analysis with the software SPSS 19.0 (IBM SPSS, Armonk, NY) and R 3.6.1. Statistical significance was set at *p* < 0.05.

## 3. Results

### 3.1. Clinical Characteristics

These patients aged from 27 to 78 years old (mean age: 55.1 years old). The 2018 International Federation of Gynecology and Obstetrics (FIGO) staging system [17] was used to implement the staging. Eleven patients were at stage IIA, 30 at stage IIB, one at stage IIIA, seven at stage IIIB, 36 at stage IIIC1r, two at stage IIIC2r, and six at stage IVA. The clinical characteristics of patients in the CR and PR groups are listed in Table 1. One month after CCRT, there were 58 patients in the CR group and 35 in the PR group. There were no significant differences in age, squamous cell carcinoma antigen (SCC), tumor size, FIGO stage, lymph node status, and histological type between the two groups (*p* > 0.05).

### 3.2. Predictive Performance of IVIM-DWI Parameters

The values of ADC, *D*, *D*^*∗*^, and *f* were markedly different between the two groups (*p* < 0.05, Table 2). Based on these parameters, the multiple logistic regression analysis was conducted and predicting model was constructed. Multivariate analysis indicated that *D* (OR = 4.029, 95%CI: 1.373–11.824, *p*=0.011) and *f* (OR = 0.889, 95%CI: 0.795–0.994, *p*=0.038) were independent differentiators with statistical significance of CR from PR of cervical cancer after CCRT. The two radiologists made a good interobserver agreement regarding IVIM-DWI parameters, with ICCs ranging from 0.83 to 0.95.

### 3.3. Predictive Performance of Texture Analysis

Significant differences were observed in nine texture features (histogram-variance, histogram-90th percentile, GLCM-correlation, GLCM-IMC1, GLCM-IMC2, GLRLM-LRE, GLRLM-RP, GLRLM-RE, and GLSZM-ZE) for discriminating CR from PR (*p* < 0.05). Multivariate analysis involving the texture features indicated that GLCM-correlation (OR = 43.789, 95%CI: 1.450–1322.227, *p*=0.030), GLRLM-LRE (OR = 9.774, 95%CI: 1.375–60.457, *p*=0.023), and GLSZM-ZE (OR = 23.738, 95%CI: 1.276–441.707, *p*=0.034) were independent differentiators with statistical significance of CR from CR of cervical cancer after CCRT (Table 3). The interobserver reproducibility indicated that ICC values ranged from 0.84 to 0.93. The result suggested favorable interobserver reproducibility of feature extraction.

### 3.4. Development and Performance of the Nomogram

**Figure 2:**
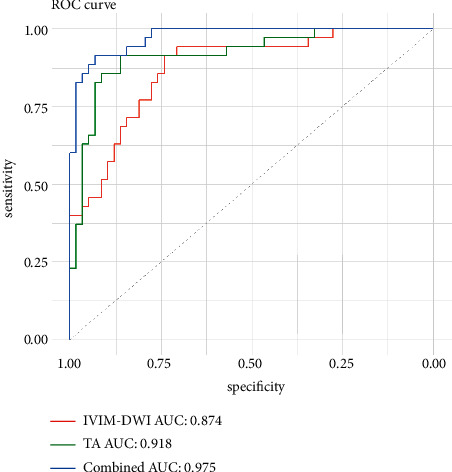
ROC curves of IVIM-DWI, TA, and IVIM-DWI with TA to distinguish CR from PR of cervical cancer after CCRT. IVIM-DWI: intravoxel incoherent motion diffusion weighted imaging; TA: texture analysis; ROC: receiver operating characteristic; AUC: area under curve; CR: complete response; PR: partial response.

**Figure 3:**
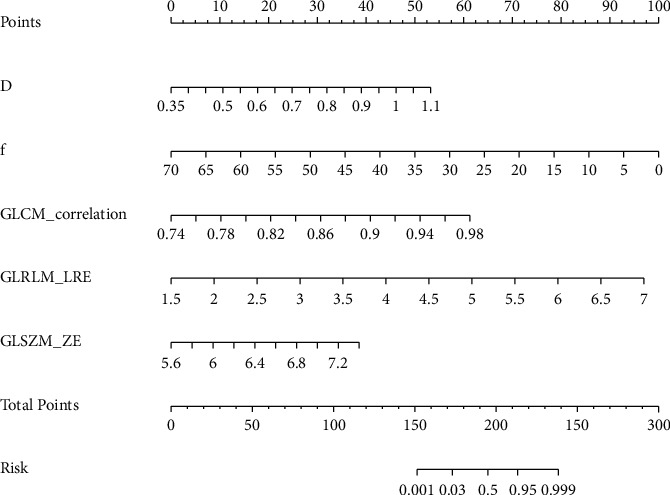
A regression coefficient-based nomogram to predict the possibility of residual tumor after concurrent chemotherapy for one month. A line is drawn perpendicular from the one axis of a parameter to the top line marked with “points.” The number of points is counted for all parameters. Then, a line is drawn from the axis marked with “total points” to the bottom line to determine the possibility of residual tumor of cervical cancer. *D*: slow diffusion coefficient; *f*: perfusion-related diffusion fraction; GLCM: gray level co-occurrence matrix; GLRLM: gray level run length matrix; GLSZM: gray level size zone matrix; LRE: long run emphasis; ZE: zone entropy.

**Figure 4:**
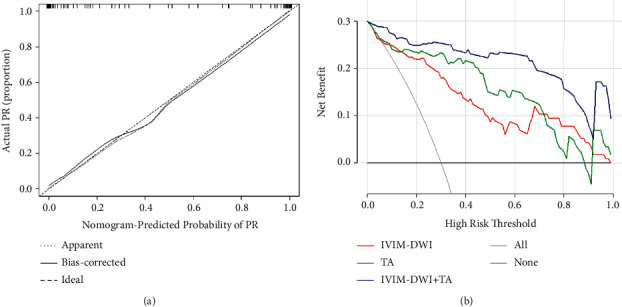
(a) The fivefold cross-validation plot of the nomogram. Brier score = 0.060. C-index = 0.953. The 45° dashed line refers to the location of an ideal nomogram, with identical actual and predicted probabilities. The dotted line refers to the performance of the apparent nomogram, and the solid line refers to the performance of the nomogram with bootstrap correction. (b) Decision curve analysis (DCA). *y*-axis indicates the net benefit, and *x*-axis indicates the threshold probability. Compared with the IVIM-DWI (red line), TA (green line), treat-all strategy (gray line), and treat-none strategy (horizontal black line), the combination of IVIM-DWI combined with TA nomogram (blue line) achieves the highest net benefit. IVIM-DWI: intravoxel incoherent motion diffusion weighted imaging; TA: texture analysis.

The ROC curves of IVIM-DWI, TA, and IVIM-DWI combined with TA models are illustrated in Figure 2, and the corresponding values of AUC, accuracy, specificity, and sensitivity are listed in Table 4. To differentiate CR from PR, the combination of IVIM-DWI and TA had the highest AUC (0.975, 95%CI: 0.950–1.000), accuracy (92.5%), specificity (91.4%), and sensitivity (93.1%). Therefore, a nomogram was developed to predict the possibility of residual tumor after one month of CCRT for cervical cancer (Figure 3). The nomogram was based on IVIM-DWI parameters (*D*, *f*) and texture features (GLCM-correlation, GLRLM-LRE, and GLSZM-ZE). For each factor, a weighted number of points were assigned. For each patient, the total number of points was calculated with the nomogram, which could also be used to estimate the probability of residual tumor. The nomogram calibration curve suggested a good agreement between observation and prediction (Figure 4(a)). Brier score was 0.060, and C-index was 0.953. Finally, we used decision curve analysis (DCA) to determine whether the nomogram would assist in implementing clinical treatment strategies (Figure 4(b)). The decision curve indicated that the nomogram of IVIM-DWI combined with TA had relatively good performance. DCA, across the range of reasonable threshold probabilities, indicated that the model of IVIM-DWI combined with TA had the most net benefit, as compared with the “treat none” strategy, “treat all” strategy, IVIM-DWI model, and TA model.

## 4. Discussion

As early occult symptoms, most cervical cancer patients are diagnosed with locally advanced cervical cancer at the time of treatment [18]. Therefore, CCRT is mainly recommended as the most proper therapeutic option. Herein, we employed IVIM-DWI parameters and texture features to predict the early treatment response in cases with cervical cancer undergoing CCRT. We found that *D* and *f* (IVIM-DWI parameters), as well as GLCM-correlation, GLRLM-LRE and GLSZM-ZE (texture features) were independent predictors. Consequently, we attempted to develop and validate a nomogram using IVIM-DWI parameters and texture features with satisfactory predictive efficacy.

The study revealed that lower *D* and higher *f* were remarkably related to the incidence of residual tumor, complying with the findings of previous studies [19]. *D* value indicates the true apparent diffusion value of water molecules, excluding the influence of blood perfusion, reflecting the density of cervical cancer cells, thereby indirectly reflecting the degree of differentiation [20]. In poorly differentiated cervical cancer, a higher cell proliferation rate, higher number of cells, and smaller extracellular space may result in a lower *D* value in CR group [21]. This may indicate that tumors in the CR group have richer microvascular formation, stronger blood supply, and higher oxygen content, leading to greater sensitivity of tumor tissue to CCRT. *f* refers to the blood volume fraction of voxel, which could demonstrate the capillary density, and indirectly shows blood flow perfusion in tissues. As for poorly differentiated tumors, the higher *f* value may be caused by the stronger blood supply, which could deliver more chemotherapeutic drugs to the tumor tissues and promote tumor regression [20]. We further confirmed that the *D* and *f* values were independent predictors (OR = 4.029, *p*=0.011; OR = 0.889, *p*=0.038, resp.) in differentiating CR from PR with multiple logistic regression analysis. The AUC of *D* combined with *f* was 0.874, with an accuracy of 80.6%, specificity of 91.4%, and sensitivity of 74.1%, respectively, which may be very useful imaging markers to predict the early treatment response of cervical cancer to CCRT after one month.

In addition to assessing IVIM-DWI parameters, we also explored and found additional imaging information on MRI images. TA, as a popular quantitative image postprocessing technology in recent years, can objectively reflect the potential biological characteristics and heterogeneity of tumors because of its quantitative extraction and analysis of pixel distribution in the lesion area [22, 23]. Recently, the application of T2WI-based TA in oncologic imaging has been investigated by several groups. Vignati et al. found that some texture features obtained through T2WI are superior to ADC value in predicting the aggressiveness of prostate cancer [24]. A recent study reported that, in breast cancer undergoing neoadjuvant chemotherapy, texture features were related to pathologic complete response only at T2WI but not at dynamic contrast-enhanced (DCE) [25]. De Cecco et al. demonstrated the efficacy of using T2WI-based TA to predict the tumor response of rectal cancer with neoadjuvant chemoradiotherapy [26]. To date, no scholar has examined TA based on T2WI sequences to predict the short-term efficacy of cervical cancer. In this study, we demonstrated that GLCM-correlation, GLRLM-LRE, and GLSZM-ZE were independent predictors. Correlation measures the similarity between elements of the gray level co-occurrence matrix in the column or row or angle. The features based on the step size matrix describe the roughness or smoothness of the image, and the long run emphasis has a larger value on the smoother image [27]. Entropy measures the randomness of gray distribution. When the tumor has complete homogeneity, the entropy value is the smallest, and when the tumor heterogeneity is large, the entropy value becomes larger [28]. When applied to T2WI, the texture features in particular reflect heterogeneity in terms of cellular distribution [29]. To predict early treatment response, the TA model of this study had high AUC (0.918), accuracy (88.2%), specificity (91.4%), and sensitivity (86.2%).

The study showed that combining IVIM-DWI with TA might create new opportunities for improving the predictive abilities. A combination of IVIM-DWI with TA for predicting early treatment response got an AUC of 0.975; the sensitivity, accuracy, and specificity were improved compared with TA or IVIM-DWI. Consequently, IVIM-DWI combined with TA can significantly enhance predictive performance.

Nomograms have exhibited significant accuracy in predicting prognosis in some malignant tumors [30, 31]. Thus, we developed a nomogram for identifying cervical cancer patients with poor efficacy after CCRT, which combined with IVIM-DWI parameters (*D*, *f*) and texture features (GLCM-correlation, GLRLM-LRE, and GLSZM-ZE). The nomogram performed well in predicting early treatment response, which showed adequate prediction in the cohort (C-index = 0.953). In addition, we applied the DCA to assess whether predictive nomogram-assisted decisions can improve the outcomes. A net benefit is defined as the proportion of true positives minus the proportion of false positives, weighted by the relative harm of false-positive and false-negative results [32]. Our results indicated that the combined predictive model, with the greatest net benefits across most of the threshold probabilities in DCA, outperformed the single IVIM-DWI and TA models. Therefore, we constructed a nomogram to facilitate clinical applications combining the IVIM-DWI parameters with texture features. The scoring system can generate the probability of residual tumor in cervical cancer patients after one month of CCRT and realize the individualized prediction of the short-term efficacy of cervical cancer patients by clinicians, which is in line with the current development trend of individualized precision medicine.

There are several limitations to our study. First, it is a retrospective study, with a relatively small number of patients. Its retrospective nature inherently increases the bias risk. Second, radiologists manually carried out image segmentation, and that process might be affected by some subjective or objective factors. Third, the nomogram was developed based on data obtained from an institution with a small number of samples, so multicenter external and prospective validation studies are needed to extend the versatility of the experimental results. Although there are limitations, our study can ensure that patients receive similar treatments and consistent pre- and posttreatment MRI. There are also complete medical records.

## 5. Conclusions

In conclusion, *D* and *f* of IVIM-DWI parameters, as well as GLCM-correlation, GLRLM-LRE, and GLSZM-ZE of texture features, are independent predictors of cervical cancer after CCRT. Our study established a nomogram that, combined IVIM-DWI and TA, can be used as an individualized noninvasive tool for predicting early treatment response in cervical cancer patients after CCRT, assisting in clinical treatment decision making and helping to improve clinical outcomes.

## Figures and Tables

**Table 1:** Comparing clinicopathologic features (CR versus PR).

Characteristics	CR (*n* = 58)	PR (*n* = 35)	*p* value
Age, years	56.5 ± 11.3	53.0 ± 9.1	0.154
Pretreatment SCC (ng/ml)	5.8 ± 6.7	7.5 ± 7.3	0.339
Tumor size (cm)	4.1 ± 1.4	4.3 ± 1.3	0.598
FIGO stage			
IIA	8	3	0.347
IIB	18	12	
IIIA	1	0	
IIIB	5	2	
IIIC1r	24	12	
IIIC2r	0	2	
IVA	2	4	
Lymph node			
Negative	33	17	0.521
Positive	25	18	
Histology			
Squamous	55	31	0.604
Adenocarcinoma	2	3	
Adenosquamous	1	0	

CR: complete response; PR: partial response; SCC: squamous cell carcinoma antigen; FIGO: international federation of gynecology and obstetrics.

**Table 2:** Statistical analysis results of the IVIM-DWI parameters in cervical cancer patients (CR versus PR).

Features	CR	PR	Univariate analysis (*p*)	Multivariate logistic regression analysis		
				OR	95%CI	*p*
ADC (×10^−3^ mm^2^/s)	0.768 ± 0.107	0.841 ± 0.110	0.004			
*D* (×10^−3^ mm^2^/s)	0.591 ± 0.105	0.744 ± 0.111	<0.001	4.029^*a*^	1.373–11.824	0.011
*D* ^*∗*^ (×10^−3^ mm^2^/s)	23.25 ± 44.28	29.50 ± 19.93	0.004			
*f* (%)	27.3 ± 12.4	15.7 ± 5.8	<0.001	0.889	0.795–0.994	0.038

^a^ per 0.1 increment. IVIM-DWI: intravoxel incoherent motion diffusion weighted imaging; CR: complete response; PR: partial response; OR: odds ratio; CI: confidence interval; ADC: apparent diffusion coefficient; *D*: slow diffusion coefficient; *D*^*∗*^: fast diffusion coefficient; *f*: perfusion-related diffusion fraction.

**Table 3:** Statistical analysis results of the texture features in cervical cancer patients (CR versus PR).

Features	CR	PR	Univariate analysis (*p*)	Multivariate logistic regression analysis		
				OR	95%CI	*p*
Histogram-90th percentile	772.57 ± 92.85	713.70 ± 108.01	0.007			
Histogram-variance	6610.00 ± 3339.54	5216.36 ± 2490.30	0.021			
GLCM-correlation	0.86 ± 0.04	0.90 ± 0.03	<0.001	43.789^*a*^	1.450–1322.227	0.030
GLCM-IMC1	−0.34 ± 0.04	−0.36 ± 0.04	0.013			
GLCM-IMC2	0.94 ± 0.02	0.95 ± 0.02	0.006			
GLRLM-LRE	3.27 ± 0.62	4.15 ± 0.87	<0.001	9.774	1.375–60.457	0.023
GLRLM-RP	0.64 ± 0.06	0.67 ± 0.05	0.008			
GLRLM-RE	4.84 ± 0.26	5.10 ± 0.29	<0.001			
GLSZM-ZE	6.47 ± 0.36	6.83 ± 0.27	<0.001	23.738	1.276–441.707	0.034

^a^per 0.1 increment. CR: complete response; PR: partial response; OR: odds ratio; CI: confidence interval; GLCM: gray level co-occurrence matrix; GLRLM: gray level run length matrix; GLSZM: gray level size zone matrix; IMC: informational measure of correlation; LRE: long run emphasis; RP: run percentage; RE: run entropy; ZE: zone entropy.

**Table 4:** The ROC analysis for IVIM-DWI, TA, and IVIM-DWI with TA.

Features	AUC	95%CI	Accuracy (%)	Sensitivity (%)	Specificity (%)	PPV (%)	NPV (%)
Overall IVIM-DWI	0.874	0.802–0.947	80.6	91.4	74.1	68.1	93.5
*D*	0.848	0.768–0.927	78.5	77.1	79.3	69.2	85.2
*f*	0.825	0.740–0.910	79.6	80.0	79.3	70.0	86.8
Overall TA	0.918	0.857–0.980	88.2	91.4	86.2	80.0	94.4
GLCM-correlation	0.744	0.645–0.843	72.0	60.0	81.0	64.5	75.8
GLRLM-LRE	0.809	0.721–0.897	77.4	74.3	79.3	68.4	83.6
GLSZM-ZE	0.777	0.683–0.870	69.9	85.7	60.3	56.6	87.5
IVIM-DWI with TA	0.975	0.950–1.000	92.5	91.4	93.1	88.9	94.7

IVIM-DWI: intravoxel incoherent motion diffusion weighted imaging; TA: texture analysis; ROC: receiver operating characteristic; AUC: area under curve; CI: confidence interval; *D*: slow diffusion coefficient; *f*: perfusion-related diffusion fraction; GLCM: gray level co-occurrence matrix; GLRLM: gray level run length matrix; GLSZM: gray level size zone matrix; LRE: long run emphasis; ZE: zone entro.

## Data Availability

The data used to support the findings of this study are available from the corresponding author upon request.

## Supplementary Materials

Supplementary Materials Supplementary Table 1 lists the texture features in this study. Supplementary Figure 1 presents flow diagram of enrolled patients.
