# Supplementary material for: Combining Intravoxel Incoherent Motion Diffusion Weighted Imaging and Texture Analysis for a Nomogram to Predict Early Treatment Response to Concurrent Chemoradiotherapy in Cervical Cancer Patients
Source: J Oncol. 2021 Dec 24;2021:9345353. doi: 10.1155/2021/9345353 (PMC8720018; doi:10.1155/2021/9345353)
Supplement: Supplementary Materials — Supplementary Table 1 lists the texture features in this study. Supplementary Figure 1 presents flow diagram of enrolled patients. [file 9345353.f1.docx]

**Supplementary material**

Supplementary Table S1: Texture features in this study

| Method | Features | Number of Features |
| --- | --- | --- |
| Histogram | Energy, Total Energy, Entropy, Minimum, 10th Percentile, 90th Percentile, Maximum, Mean, Median, Interquartile Range, Range, Mean Absolute Deviation (MAD), Robust Mean Absolute Deviation (RMAD), Root Mean Squared (RMS), Skewness, Kurtosis, Variance, Uniformity | 18 |
| GLCM | Autocorrelation, Joint Average, Cluster Prominence, Cluster Shade, Cluster Tendency, Contrast, Correlation, Difference Average, Difference Entropy, Difference Variance, Joint Energy, Joint Entropy, Informational Measure of Correlation (IMC) 1, Informational Measure of Correlation (IMC) 2, Inverse Difference Moment (IDM), Maximal Correlation Coefficient (MCC), Inverse Difference Moment Normalized (IDMN), Inverse Difference (ID), Inverse Difference Normalized (IDN), Inverse Variance, Maximum Probability, Sum Average, Sum Entropy, Sum of Squares | 24 |
| GLRLM | Short Run Emphasis (SRE), Long Run Emphasis (LRE), Gray Level Non-Uniformity (GLN), Gray Level Non-Uniformity Normalized (GLNN), Run Length Non-Uniformity (RLN), Run Length Non-Uniformity Normalized (RLNN), Run Percentage (RP), Gray Level Variance (GLV), Run Variance (RV), Run Entropy (RE), Low Gray Level Run Emphasis (LGLRE), High Gray Level Run Emphasis (HGLRE), Short Run Low Gray Level Emphasis (SRLGLE), Short Run High Gray Level Emphasis (SRHGLE), Long Run Low Gray Level Emphasis (LRLGLE), Long Run High Gray Level Emphasis (LRHGLE) | 16 |
| GLSZM | Small Area Emphasis (SAE), Large Area Emphasis (LAE), Gray Level Non-Uniformity (GLN), Gray Level Non-Uniformity Normalized (GLNN), Size-Zone Non-Uniformity (SZN), Size-Zone Non-Uniformity Normalized (SZNN), Zone Percentage (ZP), Gray Level Variance (GLV), Zone Variance (ZV), Zone Entropy (ZE), Low Gray Level Zone Emphasis (LGLZE), High Gray Level Zone Emphasis (HGLZE), Small Area Low Gray Level Emphasis (SALGLE), Large Area Low Gray Level Emphasis (LALGLE), Large Area High Gray Level Emphasis (LAHGLE) | 16 |

GLCM, gray level cooccurrence matrix; GLRLM, gray level run length matrix; GLSZM, gray level size zone matrix

**
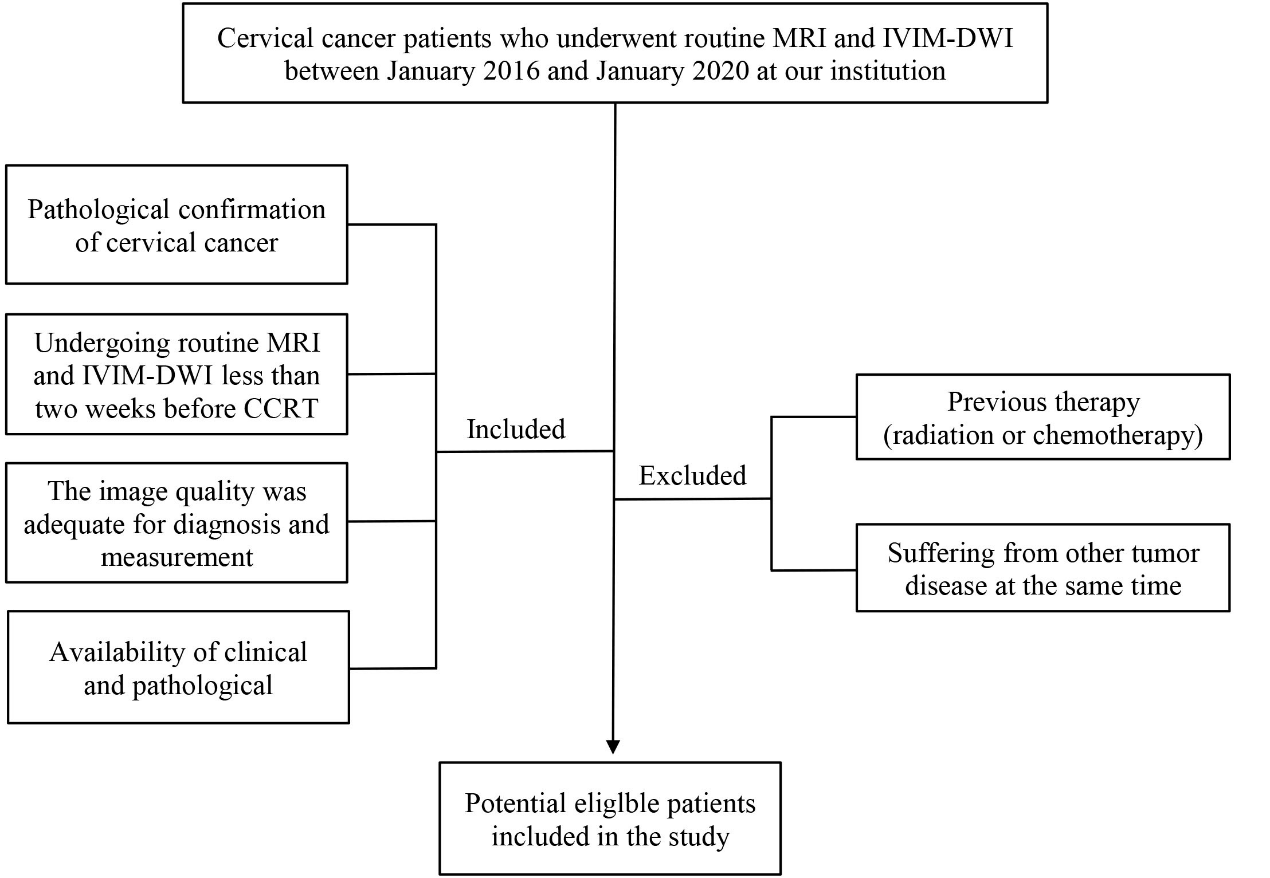
**

Supplementary Figure 1: Flow diagram of enrolled patients. MRI, magnetic resonance imaging; IVIM-DWI, intravoxel incoherent motion diffusion weighted imaging; CCRT, concurrent chemoradiotherapy.
